# Estrogen-Related Receptor Alpha Confers Methotrexate Resistance via Attenuation of Reactive Oxygen Species Production and P53 Mediated Apoptosis in Osteosarcoma Cells

**DOI:** 10.1155/2014/616025

**Published:** 2014-05-19

**Authors:** Peng Chen, Haibin Wang, Zhijian Duan, June X. Zou, Hongwu Chen, Wei He, Junjian Wang

**Affiliations:** ^1^First School of Clinical Medicine, Guangzhou University of Chinese Medicine, Guangzhou 510405, China; ^2^Cancer Center, University of California, Davis, Sacramento, CA 95817, USA; ^3^State Key Hip Joints Center, First Affiliated Hospital of Guangzhou University of Chinese Medicine, No. 12 Jichang Road, Guangzhou, Guangdong 510405, China

## Abstract

Osteosarcoma (OS) is a malignant tumor mainly occurring in children and adolescents. Methotrexate (MTX), a chemotherapy agent, is widely used in treating OS. However, treatment failures are common due to acquired chemoresistance, for which the underlying molecular mechanisms are still unclear. In this study, we report that overexpression of estrogen-related receptor alpha (ERR**α**), an orphan nuclear receptor, promoted cell survival and blocked MTX-induced cell death in U2OS cells. We showed that MTX induced ROS production in MTX-sensitive U2OS cells while ERR**α** effectively blocked the ROS production and ROS associated cell apoptosis. Our further studies demonstrated that ERR**α** suppressed ROS induction of tumor suppressor P53 and its target genes NOXA and XAF1 which are mediators of P53-dependent apoptosis. In conclusion, this study demonstrated that ERR**α** plays an important role in the development of MTX resistance through blocking MTX-induced ROS production and attenuating the activation of p53 mediated apoptosis signaling pathway, and points to ERR**α** as a novel target for improving osteosarcoma therapy.

## 1. Introduction

Osteosarcoma (OS) is one of the most common forms of childhood and adolescent cancer, comprising approximately 20% of all primary bone cancers, which is the second leading cause of mortality in this age group [1, 2]. Currently, the use of neoadjuvant chemotherapy combined with surgical resection is the mainstay therapy in OS [3]. The antifolate, chemotherapeutic agent methotrexate (MTX), is widely used to treat OS. However, approximately 30% of the OS patients suffer the recurrent or metastatic diseases due to the development of drug resistance [4]. Therefore, it is imperative to understand the molecular mechanisms underlying the MTX resistance and identify novel treatment strategies for this disease.

Estrogen-related receptors alpha (ERR*α*) is an orphan nuclear receptor that plays a key role in regulating metabolic processes. ERR*α* is abundantly expressed in tissues with high-energy demand such as heart, kidney, skeletal muscle, and brown adipose tissues [5, 6]. In addition to its role in the control of energy metabolic processes, there is cumulative evidence showing potential functions of ERR*α* in cancer development and progress. ERR*α* overexpression is associated with poor clinical outcomes not only in breast, ovarian, and prostate cancers, but also in nonendocrine related colon cancer [7–10]. Our previous reports have shown that ERR*α* suppression efficiently induced cancer cell death via induction of reactive oxygen species (ROS) production [11, 12]. ROS are chemically reactive molecules and have important roles in cell signaling and homeostasis [13]. ROS level can be dramatically increased as a response to different stress conditions, which can lead to several biological effects, ranging from alterations in cell signaling pathway and gene expression to mutagenesis, mitogenesis, or apoptosis [14, 15]. Recent studies demonstrated that chemotherapeutic drugs including MTX can induce cell death by promoting ROS production [16, 17]. We thus hypothesized that ERR*α* may be involved in chemotherapy resistance in osteosarcoma.

In this study, we demonstrated that elevated ERR*α* can result in the development of MTX resistance through blocking MTX-induced ROS production and attenuating p53-dependent apoptosis in osteosarcoma cells. Our study suggests ERR*α* is a novel target for improving osteosarcoma therapy.

## 2. Materials and Methods

### 2.1. Reagents and Antibodies

Methotrexate (MTX), N-acetylcysteine (NAC), and hydrogen peroxide (H_2_O_2_) were purchased from Sigma Chemical Company (St. Louis, MO, USA). For lentivirus generation, pMD2.G (envelope plasmid) and psPAX2 (packaging plasmid) vectors from Addgene (Cambridge, MA, USA) were used. P53 antibody was purchased from Biochem; cleaved PARP and cleaved caspase 7 antibodies were from Cell Signaling Technology (Boston, MA, USA).

### 2.2. Cell Culture and Generation of Stable Sublines

U2OS parental cells and human embryonic kidney (HEK 293T) cells were cultured in Dulbecco's Modified Essential Medium (DMEM) (Gibco, Grand Island, NY, USA) supplemented with 1% penicillin/streptomycin and 10% fetal bovine serum (Gemini Bio Products, West Sacramento, CA, USA) at 37°C under 5% CO_2_.

To generate ERR*α* overexpression U2OS stable sublines, full length cDNA of ERR*α* was cloned into the expression vector pLenti4/V5-DEST (Invitrogen). Lentiviruses were produced from these constructs using a three-plasmid packaging system as described [18]. U2OS cells were infected with the lentiviruses and selected with blasticidin (2.5 *μ*g/mL, Invivogen, San Diego, CA) for 4–6 weeks. Individual blasticidin-resistant clones were isolated and assayed for ERR*α* expression by immunoblotting. Clones homogeneously expressing ERR*α* were maintained in DMEM supplemented with 1% penicillin/streptomycin and 10% fetal bovine serum and 1 *μ*g/mL blasticidin at 37°C under 5% CO_2_. All cell lines were used within 20 passages.

### 2.3. Cell Survival Rate Assay

Cells in logarithmic growth phase were seeded in 6-well plates at a density of 100,000 cells per well and treated with different concentrations of MTX (0-1 *μ*M). Cells were harvested after 24, 48, and 72 hours and counted by Beckman Z1 Coulter Counter. Each treatment was performed in triplicate wells at least 3 times.

### 2.4. Trypan Blue Exclusion Assay

Cells in logarithmic growth phase were seeded in 6-well plates at a density of 100,000 cells per well and treated with different concentrations of MTX (0-1 *μ*M). Cells were harvested after 24, 48, and 72 hours and counted under a light microscope after trypan blue exclusion (0.4% trypan blue, Life Technologies, USA) according to instructions of manufacturer. Each treatment was performed in triplicate wells per experiment.

### 2.5. ROS Level

The production of intracellular ROS was measured using a 6-corboxy-2′,7′-dichlorodihydrofluorescein diacetate (DCFH-DA) fluorescent probe (Invitrogen, USA). The DCFH-DA passively enters the cell where it reacts with ROS to form the highly fluorescent compound dichlorofluorescein (DCF). In brief, cells were plated in 6-well plates and allowed to attach overnight. After treatment with the indicated concentrations of MTX for 24 h, the cells were washed 2 times with DMEM without FBS and then loaded with 10 *μ*M DCFHDA at 37°C, 5% CO_2_ for 20 min. Cells were washed another three times by PBS and then observed under a fluorescene microscope. ROS was also measured as previously described [19]. The cells were lysed using 1% SDS and sonicated. After 5 min centrifuge (14,000 rpm), supernatant was aliquoted (100 mL) into 96-well plate and measured by Victor 3 microplate reader. Signal intensity was normalized to protein concentration.

### 2.6. Quantitative RT-PCR

Total RNA was prepared using TRIzol reagent (Invitrogen, Carlsbad, CA, USA) according to the manufacturer's instructions. Total RNA (2 µg) was used to synthesize the first strand of cDNA. Quantitative RT-PCR (qRT-PCR) was performed in a 96-well Bio-Rad CFX96 real time PCR system (Bio-Rad Inc., Hercules, CA) and SYBR Premix Ex Taq kit (Perfect Real Time) (Takara Bio Inc., Shiga, Japan). *β*-Actin was applied as the inner control. Results are presented as 2^−ΔΔCT^ values, defined as the threshold PCR cycle number at which an amplified product is first detected. The 2^−ΔΔCT^ was determined as the mean of the triplicate CT values for target gene minus the mean of the triplicate CT values for *β*-actin. The primers used were P53, forward 5′-CACGCCTGTAATCCCAGCTACTC-3′ and reverse 5′-GCAATGGCACAATCTCGGCTCACT-3′; NOXA, forward 5′-CTTGGAAACGGAGTGGAA-3′ and reverse 5′-CGCCCAGTTAATCACAGGT-3′; XIAF, forward 5′-GCCTGCAAGAAACGAAACTC-3′ and reverse 5′-CTGGCCTCATGGCCTTAT-3′; *β*-actin, forward 5′-CCCAGCCATGTACGTTGCTA-3′ and reverse 5′-AGGGCATACCCCTCGTAGATG-3′.

### 2.7. Western Blotting Analysis

Cell lysates were prepared with lysis buffer. The lysates were cleared by centrifugation and total protein concentration was measured using bicinchoninic acid assay kit (Bio-Rad Laboratories). The protein samples were separated on SDS-PAGE at 10–15% and transferred to a polyvinylidene difluoride membrane (Immobilon-P transfer membrane; Millipore) with a Bio-Rad Trans-Blot semidry transfer cell apparatus. The membranes were blocked by 5% nonfat milk in Tris-buffered saline Tween-20 (TBST, pH 7.6) for 90 min at room temperature. Primary antibodies were diluted in 5% BSA of TBST and incubated for 2 h at room temperature or overnight at 4°C. After washing 3 times with TBST, the membranes were incubated with anti-rabbit, anti-mouse, or anti-goat (1/2,000; R&D Systems) secondary antibodies coupled to horseradish peroxidase for 1 h at room temperature. The membranes were washed 3 times with TBST; then the bands were detected using electrochemiluminescence (ECL) (GE Healthcare UK Ltd.).

### 2.8. Statistical Analysis

Data were expressed as means ± SD. All experiments were independently repeated at least three times. The statistical significance of differences was determined by Student's two-tailed *t*-test. *P* < 0.05 was considered statistically significant. Asterisks indicate the level of significance. The data were analyzed using SPSS 17.0.

## 3. Results

### 3.1. ERR*α* Overexpression Blocks MTX-Induced Osteosarcoma Cell Death

**Figure 1:**
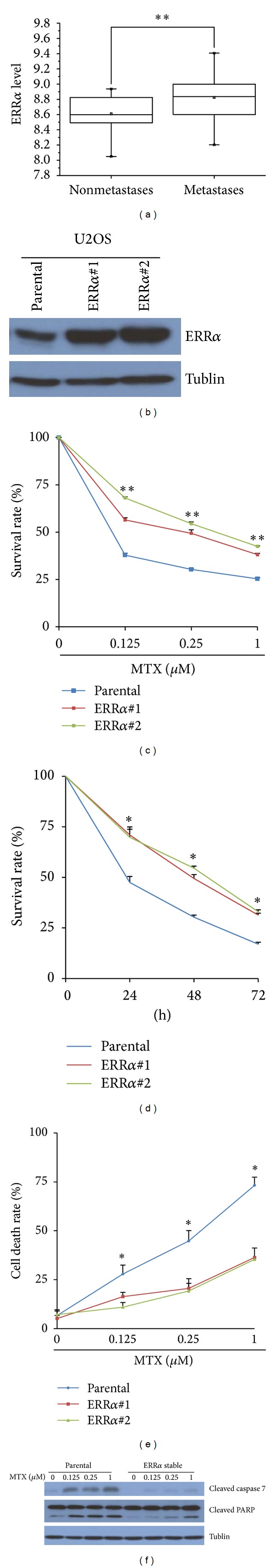
Enhanced ERR*α* increases resistance to MTX-induced cell death. (a) The expression of the ERR*α* gene was evaluated from nonmetastasis (*n* = 19) and metastasis (*n* = 34) osteosarcoma in the GEO dataset GSE21257. (b) Western blotting of ERR*α* gene in U2OS parental and U2OS ERR*α* overexpression stable cells (U2OS-ERR*α*). (c) U2OS parental and U2OS-ERR*α* (numbers 1 and 2) were treated with MTX (0-1 *μ*M) for 48 hours and then survival rate was analyzed by counting cell numbers. (d) U2OS parental and U2OS-ERR*α* cells (numbers 1 and 2) were treated with 0.25 *μ*M MTX and were collected at 24 hours, 48 hours, and 72 hours. The survival rate was analyzed by counting cell numbers. (e) U2OS parental and U2OS-ERR*α* cells (numbers 1 and 2) were treated with MTX (0-1 *μ*M) for 72 hours; dead cells were assessed by the trypan blue exclusion assay. (f) U2OS parental and U2OS-ERR*α* cells (numbers 1 and 2) were treated with MTX (0-1 *μ*M) for 48 hours and then cleaved caspase 7 and cleaved PARP were detected by Western blotting. Data are expressed as mean ± SD (*n* = 3) and analyzed using Student's *t*-test. Asterisks indicate significant differences: *P < 0.05, **P < 0.01.

To investigate the potential function of ERR*α* in osteosarcoma (OS) progression, we analyzed ERR*α* gene expression in Gene Expression Omnibus (GEO) datasets. We found that ERR*α* expression was significantly upregulated in the metastatic osteosarcomas compared with nonmetastatic tumors in GSE21257 dataset (Figure 1(a)). Metastatic osteosarcomas that are resistant to conventional chemotherapy are the major cause of death. To explore the potential role of ERR*α* in chemotherapeutic resistance, we first established U2OS stable sublines that overexpress ERR*α* (U2OS-ERR*α* number 1 and U2OS-ERR*α* number 2) (Figure 1(b)). We then examined the effect of ERR*α* overexpression on the sensitivities of U2OS cells to chemotherapeutic agent MTX. Results in Figures 1(c) and 1(d) demonstrated that overexpression of ERR*α* strongly protected the cells from MTX-induced growth inhibition. By trypan blue exclusion assay, we found that the elevated ERR*α* can efficiently block MTX-induced cell death (Figure 1(e)). As shown in Figure 1(f), MTX at a low concentration (0.125 *μ*M) effectively induced apoptosis in the parental U2OS cells, as detected by cleaved PARR and cleaved caspase 7. However, in U2OS-ERR*α* cells, MTX even at 8-fold higher concentration (1 *μ*M) did not efficiently cause cell apoptosis. These results suggested that ERR*α* conferred resistance to MTX in human osteosarcoma cells through inhibition of apoptosis.

### 3.2. MTX Induces Cell Death by Increasing ROS Production in Osteosarcoma Cells

**Figure 2:**
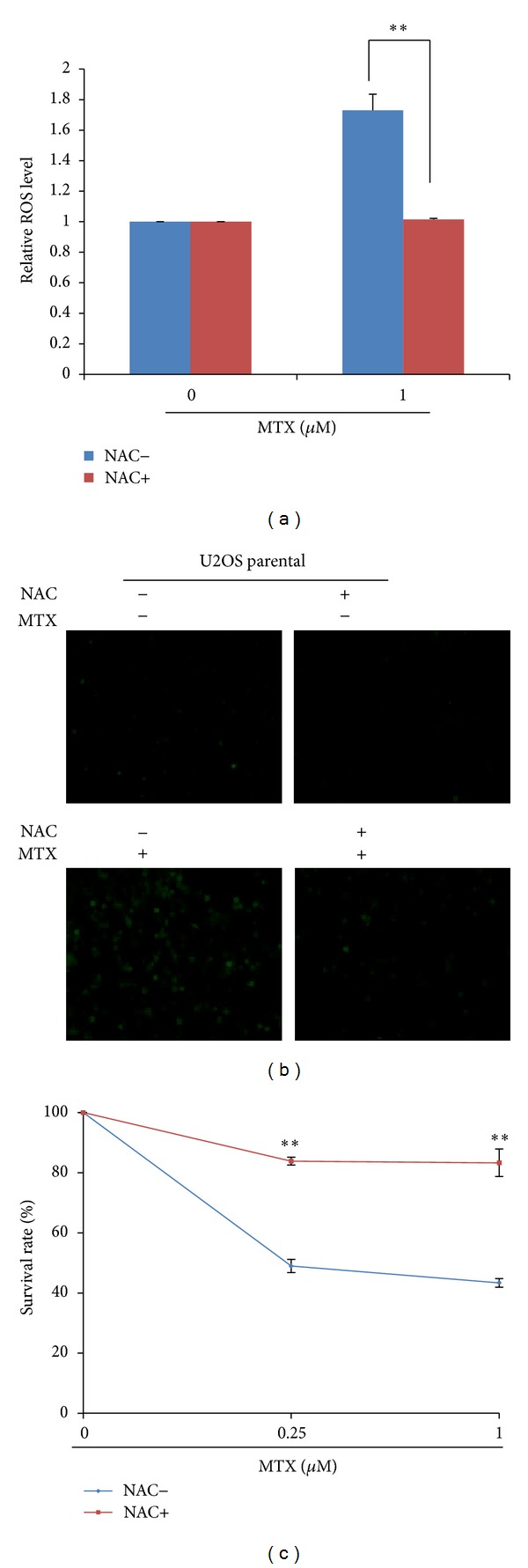
MTX induces cell death by increasing ROS production in osteosarcoma cells. ((a)-(b)) U2OS cells were treated with 1 *μ*M MTX for 24 hours; ROS production was analyzed by DCFH-DA staining using microreader and fluorescent microscope. (c) U2OS cells were pretreated with 25 mM NAC for 2 hours followed by treatment with 1 *μ*M MTX for 24 hours. ROS production was analyzed by DCFH-DA staining using microreader and fluorescent microscope. Data are expressed as mean ± SD (*n* = 3) and analyzed using Student's *t*-test. Asterisks indicate significant differences: *P < 0.05, **P < 0.01.

A number of studies indicated that reactive oxygen species (ROS) are involved in a variety of different cellular processes ranging from apoptosis and necrosis to cell proliferation and carcinogenesis [20, 21]. To determine whether ROS is involved in MTX-induced cell death, we stained the U2OS cells with CM2-DCFHDA staining and found that MTX treatment significantly increased ROS production in U2OS cells while N-acetylcysteine (NAC), an antioxidant compound, effectively blocked its induction of ROS (Figures 2(a) and 2(b)). Furthermore, simultaneous treatment with NAC almost completely protected the cells from MTX-induced cell death (Figure 2(c)). Therefore, ROS plays a critical role in MTX-induced cell death.

### 3.3. ERR*α* Confers MTX Resistance by Modulating ROS Production in Osteosarcoma Cells

**Figure 3:**
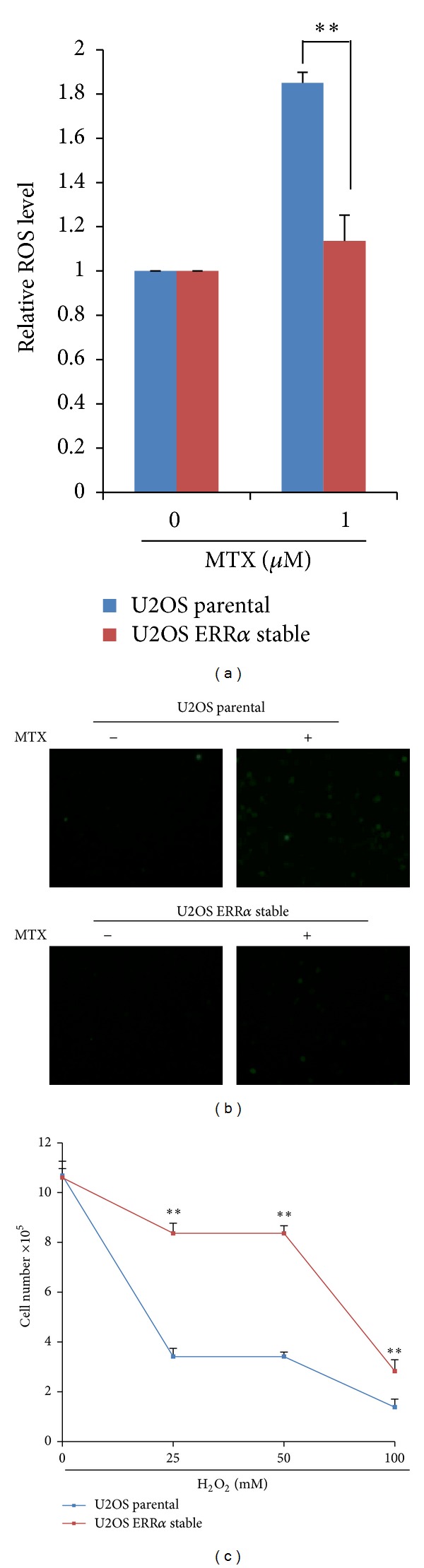
ERR*α* confers MTX resistance by modulating ROS production in osteosarcoma cells. ((a)-(b)) U2OS parental and U2OS-ERR*α* cells (numbers 1 and 2) were treated with 1 *μ*M MTX for 24 hours and then ROS production was analyzed by DCFH-DA staining using microreader and fluorescent microscope. (c) U2OS parental and U2OS-ERR*α* cells (numbers 1 and 2) were treated with different concentrations of H_2_O_2_ (0–100 mM) for 24 hours; cell numbers were counted. Data are expressed as mean ± SD (*n* = 3) and analyzed using Student's *t*-test. Asterisks indicate significant differences: *P < 0.05, **P < 0.01.

We and others demonstrated previously that ERR*α* modulates ROS production in cancer cells [12, 22, 23]. We next examined whether ERR*α*-mediated MTX resistance was through control of ROS. Results shown in Figures 3(a) and 3(b) demonstrated that overexpression of ERR*α* could efficiently block MTX-induced ROS generation. To further demonstrate that ERR*α* protected cells from MTX-induced cell death by downregulating ROS production, we treated U2OS parental and U2OS-ERR*α* cells with hydrogen peroxide (H_2_O_2_), a highly potent form of ROS. We found that ERR*α* overexpression also strikingly protected H_2_O_2_-induced cell growth inhibition even at 50 mM of H_2_O_2_ (Figure 3(c)). These results collectively suggested that ERR*α* confers MTX resistance by suppression of ROS production in osteosarcoma cells.

### 3.4. P53-Mediated Apoptosis Pathway Is Involved in ERR*α* Mediated MTX Resistance

**Figure 4:**
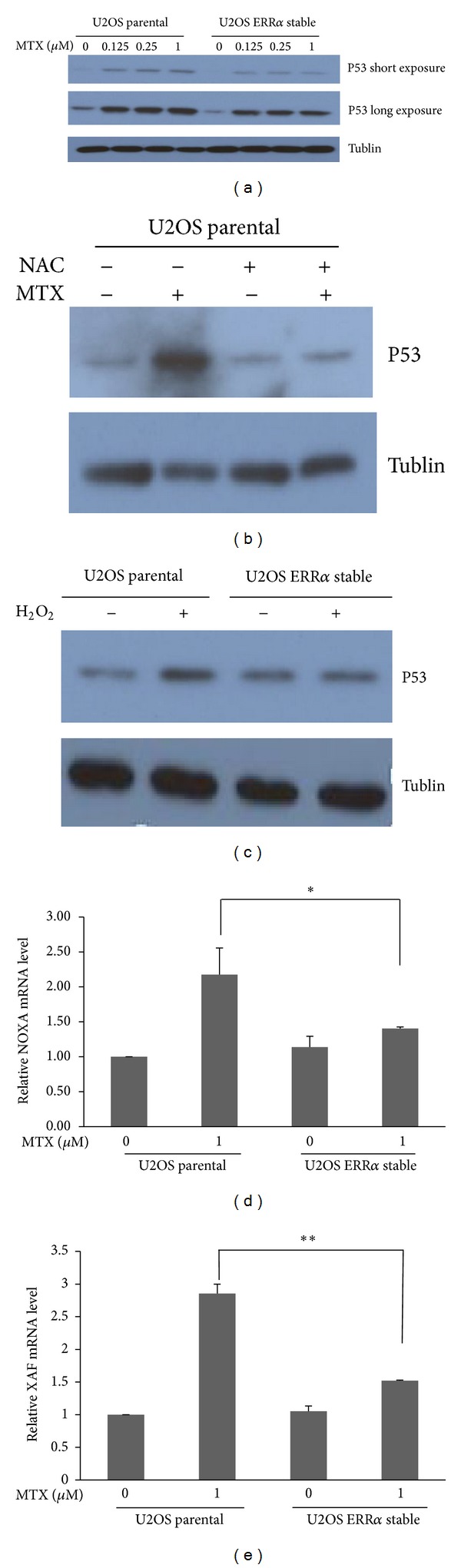
P53 apoptosis pathway is involved in ERR*α* mediated MTX resistance. (a) U2OS parental and U2OS-ERR*α* cells (numbers 1 and 2) were treated with MTX (0-1 *μ*M) for 48 hours; P53 protein level was assayed by western blotting. (b) U2OS parental cells were pretreated with 25 mM NAC for 2 hours followed by treatment with 1 *μ*M MTX for 48 hours; P53 protein level was assayed by western blotting. ((c)–(e)) U2OS parental and U2OS-ERR*α* cells (numbers 1 and 2) were treated with 1 *μ*M MTX for 48 hours, the expression of NOXA, XAF, and P53 was detected by QPCR. (f) U2OS parental and U2OS-ERR*α* cells (numbers 1 and 2) were treated with 25 mM H_2_O_2_ for 24 hours; P53 protein level was assayed by western blotting. Data are expressed as mean ± SD (*n* = 3) and analyzed using Student's *t*-test. Asterisks indicate significant differences: *P < 0.05, **P < 0.01.

It is well established that tumor suppressor P53 and its target genes are involved in ROS-induced apoptosis in different cancer cells [24, 25]. We thus examined the effect of ERR*α* overexpression and MTX on p53 protein level and the role of ROS in this process. Using Western blotting analysis, we found that MTX dose-dependently induced p53 at the protein level and that ERR*α* overexpression blocked this induction (Figure 4(a)). Consistent with the notion that MTX induction of P53 involves ROS, the antioxidant compound NAC effectively eliminated MTX-induced p53 accumulation (Figure 4(b)). Moreover, P53 induction by H_2_O_2_ could also be suppressed by ERR*α* (Figure 4(c)). Importantly ERR*α* overexpression also blocked MTX-induced NOXA and XAF1 expression which are p53 direct target genes for promoting cell apoptosis (Figures 4(d) and 4(e)). These results collectively indicated that ERR*α* overexpression suppressed MTX-induced p53 activity through modulating ROS production.

## 4. Discussion

Osteosarcoma (OS) is the most common tumor of bone. Currently, the use of neoadjuvant chemotherapy combined with surgical resection is the mainstay therapy in OS [3]. The overall survival for osteosarcoma patients has been increased to over 70%. However, the 5-year survival of patients with OS metastasis still remains about 20–30%. Drug resistance is still a significant clinical problem in effective therapy of osteosarcoma. In this study, we showed that overexpressing ERR*α* in human osteosarcoma cells promotes the survival of OS cells through inhibiting MTX-induced apoptosis. In addition, our results indicated that ERR*α* overexpression confers osteosarcoma resistance to MTX via attenuation of reactive oxygen species production and p53-mediated apoptosis pathway in osteosarcoma U2OS cells.

The primary function of ERR*α* is believed to be the regulation of energy metabolism. Additionally, ERR*α* at least has been shown to play a role in the regulation of bone formation [26, 27]. ERR*α* is expressed throughout all developmental stages from early progenitors to bone forming osteoblasts [26]. It may play a functional role in osteoblast differentiation and bone formation by regulating the expression of osteocalcin (OC), bone sialoprotein (BSP), Runx2, and alkaline phosphatase (ALP) [28, 29]. We and others have identified that osteopontin (OPN) and osteocalcin (OC) are ERR*α* direct target genes in bone [30, 31]. Interestingly, studies also showed that OPN and OC promote cancer progression in different cancer types [32, 33]. Although it is well established that OPN and OC play important roles in bone formation, their roles in osteosarcoma remain unknown. To understand the role of ERR*α* and its target genes in osteosarcoma progression, further investigations, including using animal xenograft models, are needed to be done to demonstrate the importance of ERR*α* in advanced and chemotherapy resistance osteosarcoma. In current research, we demonstrated for the first time that the expression of ERR*α* is correlated with osteosarcoma progression and that ERR*α* overexpression can mediate MTX resistance in osteosarcoma cells. Our data collectively suggested that ERR*α* may be a novel target for combating chemotherapy resistance and advanced osteosarcoma.
